# A comprehensive analysis of the Omp85/TpsB protein superfamily structural diversity, taxonomic occurrence, and evolution

**DOI:** 10.3389/fmicb.2014.00370

**Published:** 2014-07-21

**Authors:** Eva Heinz, Trevor Lithgow

**Affiliations:** ^1^Department of Microbiology, Monash UniversityMelbourne, VIC, Australia; ^2^Victorian Bioinformatics Consortium, Monash UniversityMelbourne, VIC, Australia

**Keywords:** outer membrane protein assembly, Omp85, Omp85/TpsB superfamily, two-partner secretion, BamA

## Abstract

Members of the Omp85/TpsB protein superfamily are ubiquitously distributed in Gram-negative bacteria, and function in protein translocation (e.g., FhaC) or the assembly of outer membrane proteins (e.g., BamA). Several recent findings are suggestive of a further level of variation in the superfamily, including the identification of the novel membrane protein assembly factor TamA and protein translocase PlpD. To investigate the diversity and the causal evolutionary events, we undertook a comprehensive comparative sequence analysis of the Omp85/TpsB proteins. A total of 10 protein subfamilies were apparent, distinguished in their domain structure and sequence signatures. In addition to the proteins FhaC, BamA, and TamA, for which structural and functional information is available, are families of proteins with so far undescribed domain architectures linked to the Omp85 β-barrel domain. This study brings a classification structure to a dynamic protein superfamily of high interest given its essential function for Gram-negative bacteria as well as its diverse domain architecture, and we discuss several scenarios of putative functions of these so far undescribed proteins.

## INTRODUCTION

The Omp85/TpsB protein superfamily is a unique group of bacterial outer membrane proteins, which can function as protein translocases or as membrane protein assembly factors (Mazar and Cotter, 2007; Hagan et al., 2011); with a well-studied example described for each of these two functions: The TpsB family protein FhaC secretes a partner protein (FHA) through the outer membrane to the extracellular milieu (Mazar and Cotter, 2007; Jacob-Dubuisson et al., 2013). The Omp85 family protein BamA functions as chaperone, receiving nascent β-barrel proteins from periplasmic chaperones and assembling these into the outer membrane (Hagan et al., 2011; Kim et al., 2012).

The Omp85/TpsB protein superfamily is characterized through sequence similarity and shared structural characteristics (Yen et al., 2002; Moslavac et al., 2005), there is however a clear separation between the Omp85 family (e.g., BamA) and TpsB family (e.g., FhaC) at the sequence level. This is reflected in two defining Pfam profiles: PF01103 (“Bac_surface_Ag”) for Omp85 proteins and PF03865 (“ShlB”) for TpsB proteins. Despite this distinction, there is an underlying sequence similarity in the membrane-embedded β-barrel domains (Yen et al., 2002; Moslavac et al., 2005), which is also represented on a structural level (Clantin et al., 2007; Gruss et al., 2013; Noinaj et al., 2013). In both of these proteins, a series of ∼10 kDa globular domains (Polypeptide Transport Domains or POTRAs; Sanchez-Pulido et al., 2003) stretch out from the N-terminal part of the barrel domain, and are located within the bacterial periplasm.

Differences between the two families are also found in their taxonomic distribution. TpsB proteins function as translocases dedicated to the secretion of a single protein substrate, characteristically haemagglutinin-like partner proteins, and they are therefore found predominantly in pathogenic organisms in a distribution pattern indicative of horizontal gene transfer (HGT). Conversely, the Omp85 protein BamA is essential for the assembly of β-barrel proteins, and Omp85 family proteins have been reported in all Gram-negative phyla (Cavalier-Smith, 2006; Sutcliffe, 2010; Errington, 2013). Mitochondria and plastids, as eukaryotic organelles derived from bacterial endosymbionts, each harbor an Omp85 protein in their outer membranes. These proteins are homologs of BamA, chaperoning the assembly of β-barrel proteins into organellar outer membranes. The mitochondrial Omp85 protein, Sam50, is most similar to α-proteobacterial BamA (Gentle et al., 2004) and the plastid proteins Toc75-III and Oep80 are most similar to the cyanobacterial Omp85 proteins (Bolter et al., 1998; Reumann and Keegstra, 1999; Schleiff and Becker, 2011). This correlates with our understanding of the ancestry of the organelles.

Two recent findings have highlighted the complexity of this superfamily, and insist on a refinement of the existing Omp85/TpsB dichotomy. The translocation and assembly machinery (TAM) consists of the outer membrane protein TamA and the inner membrane protein TamB (Selkrig et al., 2012), and functions in the assembly of outer membrane proteins. Structurally, TamA is similar to BamA (Gruss et al., 2013; Noinaj et al., 2013), but has only three POTRA domains and can be clearly distinguished from BamA based on sequence characteristics. A further Omp85 protein was identified recently in *Pseudomonas aeruginosa*, the patatin-like Omp85 protein PlpD, which carries a single POTRA domain followed by a patatin domain at the N-terminus. The patatin domain is translocated across the outer membrane and released into the environment, potentially acting as virulence factor for *Pseudomonas* (Salacha et al., 2010).

To understand the diversity and distribution of this important protein superfamily, we performed a comprehensive analysis, extracting all detectable Omp85/TpsB-like sequences from current databases, followed by manual curation. Clustering analysis was used to group the sequences, and further analyses were used to improve this grouping scheme. We observed 10 domain architectures; several of these so far undescribed, and we have developed a comprehensive classification scheme based around the domain structure and sequence characteristics. This classification scheme provides a framework for functional associations, and yields useful insights into the way this family of proteins has evolved. The dynamic evolutionary history of the Omp85/TpsB superfamily is reminiscent of other molecular chaperones, and the implications of these similarities are discussed.

## MATERIALS AND METHODS

### DATABASES AND SOFTWARE PACKAGES

All searches were performed against, and sequences and taxonomic information were retrieved from, the UniProt database (Magrane and Consortium, 2011; release 06032013) unless stated otherwise. Protein domains were retrieved from the Interpro database (Hunter et al., 2012; version 41.0). Markov Clustering (MCL) was performed using the mclblastline suite (mcl version 12-135; Enright et al., 2002), with several different inflation parameters, where the optimal settings were chosen after manual inspections of the resulting datasets with respect to known functionally different homologs (BamA, TamA, Sam50, Sam51); all-against-all blast values for mclblastline clustering were obtained by using the blastall -p blastp command (blastall 2.2.24) with the -m8 output option, all other settings as default. For network representations in cytoscape (version 3.1; Shannon et al., 2003), protein diversity was first reduced by clustering all sequences with the usearch program (Edgar, 2010; search performed using the –cluster_fast algorithm with a cutoff of –id 0.80, the –centroid command was used to obtain the sequences). The resulting sequences were used as input for an all-against-all blastp run (version 2.2.26+; cutoff *e-*value 1E-5) and self-loops were removed before network analyses. For clustering of the barrel or N-terminal domains only, the same accession numbers as used for the full-length clustering (i.e., the centroids resulting from uclust) were retrieved from the respective barrel-only or N-terminus-only sequence sets; the formation of these datasets is described below. Lipoprotein signature signal sequences were recovered from the LipoP predictor with default settings (version 1.0, Juncker et al., 2003), and secondary structure predictions to identify and confirm POTRA and other domains in novel Omp85 subfamilies were performed using Phyre2 (Kelley and Sternberg, 2009) and Praline (Simossis and Heringa, 2005). For clusters >100 amino acids, usearch was used as above reducing the number sequences to –id 0.50 prior to submission to Phyre2. The heatmap representation was performed with the R software package (The R Project for Statistical Computing)^1^ using the “heatmap” command with the scale set to “none,” and representation of protein structures was performed using the UCSF Chimera package (Pettersen et al., 2004).

### Omp85/TpsB SUPERFAMILY DATASET GENERATION

The initial HMMER profiles were retrieved from the Pfam website^2^ (Punta et al., 2012) as PF01103.18 and PF03865.8, and searched against UniProt. The HMMER search (version 3.1dev; Eddy, 2011) was performed with hmmsearch using an *e*-value cutoff –incE 1 for the PF01103 dataset and –incE 0.1 for the PF03865 dataset and both searches were performed by disabling all additional filters (–max option). Following manual inspections, we decided to include all hits below the inclusion cutoff for further analyses as well, as several Omp85/TpsB-like proteins were identified below the cutoff values, resulting in a combined dataset of 13,713 protein sequences after removing proteins detected by both profiles. We sought to better distinguish contaminants, which share some underlying sequence similarity with Omp85/TpsB proteins but belong to different protein families, from highly divergent Omp85/TpsB proteins. To this end, sequences were grouped into their UniProt100 groups to decrease the sample size, and clustered using the mclblastline (*e*-value cutoff of 1E-2, inflation value 1.5, scheme 7). These initial clusters were manually investigated to identify contaminants by analysing similarity of the proteins in the nr and UniProt databases, Pfam domain profiles and additional domain and other annotations as given in public databases. In any cluster containing contaminants belonging to different protein families, all proteins grouped in this cluster (including hypothetical and unknown proteins without annotated features) were considered contaminants; whereas in a cluster containing Omp85/TpsB-like proteins, all proteins (including hypothetical and unknown without annotated features) were considered Omp85/TpsB members. No contradicting clusters (being a mixture of clear contaminants and true Omp85/TpsB proteins) were encountered. After removal of all contaminants from the original search results (i.e., removal of all sequences belonging to the respective UniProt100 groups judged as contaminants), the final dataset was clustered again using mclblastline (*e*-value cutoff 1E-2, inflation value 1.3, scheme 7). A final curation step included removal of sequences with less than 250aa, and the final dataset consisted of 12,869 proteins in 40 clusters, all accession numbers for the respective clusters are given in **Table S1**. For analyses of the presence or absence of the respective copies only proteins and their corresponding taxa flagged as “complete proteome” entry in the UniProt database were considered. The taxonomic tree used to plot different numbers of paralogs and orthologs was obtained from sTOL (Fang et al., 2013)^3^, download date 30. 04. 2014. The graphical tree representation was prepared using the iTol web tool (Letunic and Bork, 2011).

### DATASET GENERATION TO ANALYZE N-TERMINI, BARREL REGIONS, AND POTRAs

For the barrel-only dataset used in the protein–protein similarity network analyses as indicated in the figure legend, all sequences were retrieved using the first position of the alignment (the “envelope start” position) as given in the initial HMMER search result as the N-terminal border of the barrel, and the actual end of the protein sequence as the C-terminal border. For proteins retrieved in both searches, the higher scoring HMMER result was used. The N-terminal dataset for all sequences was retrieved using the actual start position of the sequence as N-terminus and the first position of the HMMER search alignment region (i.e., the start of the barrel domain as described above) as C-terminus; since some subfamilies have only a very short N-terminal region, sequences with less than 20 aa remaining for the N-terminus were removed from the dataset. For the POTRA analyses, the respective main clusters (minimum 30 members) as given in **Table S1** with predicted POTRA domains (BamA, TamA, BamA-like, Patatin-like, Sam50, FhaC, Hmw1B, Lipo) were reduced to id 0.50 using uclust. These sequences were submitted to the Praline (Simossis and Heringa, 2005) web server, and the secondary structure prediction was performed with the implemented PsiPred program (McGuffin et al., 2000). The POTRA domains were subsequently extracted from the aligned id 0.50 datasets, and sequences <25 aa and >125 aa were removed. Only one set of POTRA domains per cluster was defined, removing additionally gained POTRA domains in small numbers of sequences. In addition, we extracted all FtsQ sequences available in the Swissprot database (retrieved on 12. 02. 2014 online; search term “PF03799”), extracted the POTRA domain as described above, and added it to our dataset, which was then used for clustering in cytoscape as described above with an e-value cutoff of 1E-3.

### PHYLOGENETIC TREE INFERENCE

Alignments were generated with muscle (Edgar, 2004), and sites for tree inference were chosen using trimal under the “-automated1” setting (Capella-Gutierrez et al., 2009). Trees were calculated using Phylobayes v3.3d (Lartillot et al., 2009) under the C20 or C60 model as indicated in the figure legends, with two independent chains for each, and chain convergence was analyzed manually using the bpcomp and tracecomp command as suggested by the authors (Lartillot et al., 2009), posterior probabilities are shown as branch support values.

## RESULTS

### THE Omp85/TpsB SUPERFAMILY IS COMPOSED OF 10 DISTINCT SUBFAMILIES

The defining feature of the Omp85/TpsB superfamily is the membrane-embedded barrel domain (Gentle et al., 2004; Arnold et al., 2010; Salacha et al., 2010; Selkrig et al., 2012). To find the maximal number of Omp85/TpsB proteins from which to start a classification, only the conserved regions of the barrel-domain sequences (see section “Methods”) were used as search input. By this definition, a search against the UniProt database and manual curation identified 12,869 protein sequences in bacteria and eukaryotes as members of the Omp85/TpsB superfamily (**Table S1**). No Omp85/TpsB proteins were detected in archaea.

**FIGURE 1:**
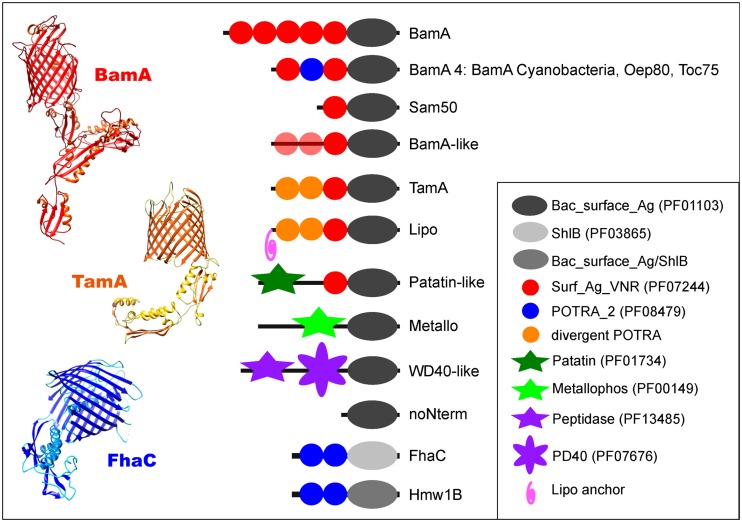
**Structural diversity of the Omp85/TpsB superfamily.** Schematic representation of the domain architectures (detailed in **Table S2**) of the ten bacterial protein subfamilies that comprise the Omp85/TpsB superfamily, as well as the eukaryotic Sam50. The cyanobacterial BamA is shown as a separate group due to its exceptional domain architecture within the BamA subfamily. Also shown are the crystal structures for the three known exemplars: BamA (PDB 4K3B; Noinaj et al., 2013), TamA (PDB 4C00; Gruss et al., 2013) and FhaC (PDB 2QDZ; Clantin et al., 2007). In each case the POTRA domains can be seen emanating from the N-terminal region of the barrel domain.

Unexpectedly, many proteins were discovered to be distinct from the known domain arrangement based on an absence of POTRA sequences in their domain profiles. The 40 clusters retrieved from our initial sequence clustering could be resolved to represent 10 protein subfamilies in bacteria (**Figure 1**). Most of these have not been recognized previously, including POTRA-containing Omp85 proteins divergent from the cognate BamA and TamA (“BamA-like”), as well as non-POTRA domain architectures described below (**Figure 1**; **Table S2**). The sequence-based split of the TpsB family into two groups (“FhaC” and “Hmw1B”) was observed as before (Jacob-Dubuisson et al., 2013), and no further subfamilies or domain profiles could be identified associated with the TpsB-type barrel domain.

**FIGURE 2:**
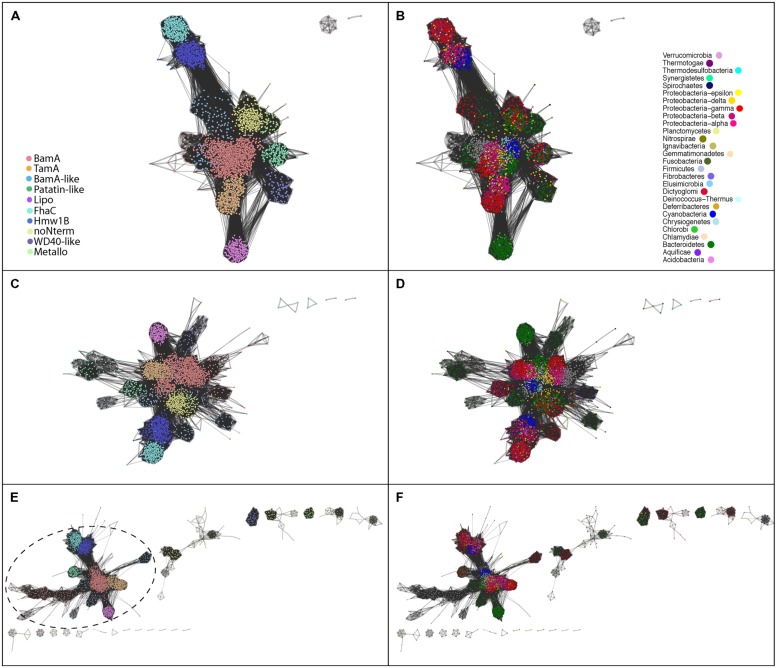
**Distinctions between the Omp85/TpsB subgroups in sequence similarities. (A)** Protein–protein similarity network representation of full-length sequences, demonstrating the ten bacterial subfamilies; due to its origin from bacterial BamA the eukaryotic sequences were included to the BamA subfamily. **(C)** The similarity network representation of barrel-domain sequences and **(E)** the similarity network representation of N-terminal domain sequences, where the colors describe the different subfamilies as depicted in **(A)**. The circled area in **(E)** illustrates a connected cluster consisting of proteins encoding one or more POTRA domains, whereas the sequences with alternative (non-POTRA) N-terminal domains segregate into distinct groups. **(B,D,F)** are a recolouring of **(A,C,E)**, respectively, according to different bacterial Phyla (eukaryotes in gray). The color corresponding to each phylum is depicted in **(B)**.

The most conservative hypothesis for the function of the unknown subfamilies with high similarity to Omp85 proteins is a role in some aspect of protein assembly into or across the outer membrane. This is the general function of Omp85 family members, but experimentation will be required to test this hypothesis. The diverse domain architectures identified in the N-terminal region of the Omp85 barrel, serve to define the ten protein subfamilies (Figures 1 and 2A).

Proteins in the WD40-Omp85 cluster have a beta-propeller-like structure encoded in the N-terminal WD40 domain repeat sequences (**Figure 1**; **Table S3**). There are two relevant WD40 domain proteins associated with the functions ascribed to the Omp85 family. The first, TolB, is a periplasmic component of the bacterial Tol-Pal system with a WD40 domain structure (Bonsor et al., 2007); the beta-propeller domain of TolB also shows the highest structural similarity to the Omp85 WD40 domain structure. A function in peptidoglycan recycling, or the covalent linking with lipoproteins, was suggested for TolB (Abergel et al., 1999) and its partner protein Pal can interact with BamA (Anwari et al., 2010). BamB is a highly conserved WD40 protein found in most Proteobacteria (Anwari et al., 2012) that serves as a lipoprotein partner of BamA (Albrecht and Zeth, 2011; Heuck et al., 2011; Kim and Paetzel, 2011; Noinaj et al., 2011). These Omp85 WD40-like proteins are therefore reminiscent of a fusion between BamA and BamB, which serves as a platform for the attachment of other members of the BAM complex.

Like the TpsB proteins and the Toc75 found in plastids, the patatin-like Omp85 protein PlpD from Pseudomonas aeruginosa translocates proteins through the outer membrane. As characterized recently, PlpD delivers a lipolytic enzyme domain onto the bacterial surface by a mechanism that was suggested to be similar to that of FhaC (Salacha et al., 2010). This is made all the more intriguing, given the close similarity between PlpD and members of the Omp85 family, rather than TpsB family, of proteins (**Figure 2C**). Structural investigations into the patatin-like Omp85 proteins will be fascinating, given that the structures of BamA and TamA both show the Omp85-type barrel domain to be fully closed to the extracellular milieu.

Depending on the final topology of the proteins, the Omp85-metalloproteases (“Metallo”) might aid in the proteolytic quality control in the periplasm as do proteases such as Clp and DegP (Merdanovic et al., 2011) or, by analogy with the action of the patatin-like Omp85 proteins, the metalloprotease domain could function as a virulence factor if translocated across the outer membrane. Theoretical support for the former hypothesis comes from observations that the specific metalloprotease domain (PF00149) found in these Omp85 proteins shows over 400 annotated domain architectures in Pfam, linking it to other domains that would be located in the periplasm/cell wall. These include domain architectures associated with periplasmic/outer envelope locations such as the peptidoglycan-binding LysM domain (PF01476), a cell-wall binding domain (PF04122), a Gram-positive anchor domain (PF00746) and S-layer domains (PF00395) all suggestive of a function in diverse different cell envelope environments.

**FIGURE 3:**
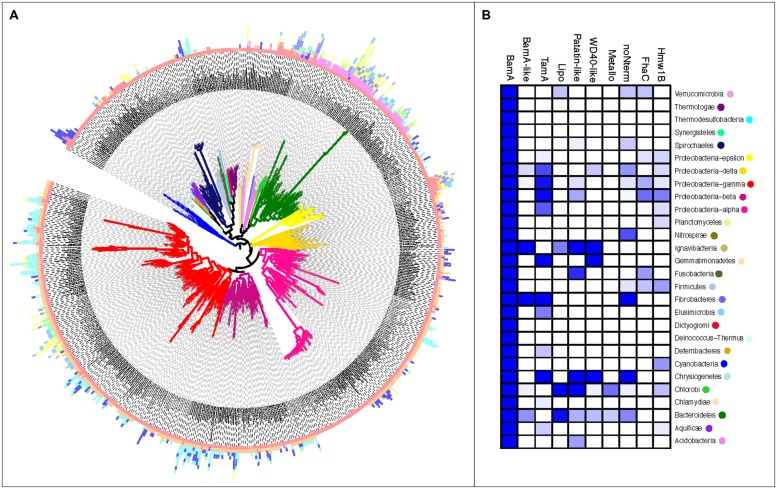
**The uneven distribution of Omp85/TpsB subgroups by taxa. (A)** Sequences of the Omp85/TpsB protein subfamilies are represented by bars plotted to the respective taxa in the guidance tree. Length of the bar indicates numbers of gene copies, bar color indicates the Omp85/TpsB subfamilies as in Figure 1, branch color indicates bacterial Phylum as displayed in Figure 1. **(B)** A heatmap indicating the percentage of completed genomes of the respective Phylum in which the respective Omp85/TpsB subfamily has been identified; colors are based on a percentage scale ranging from deep blue (100%) to white (0%).

The Omp85 lipoproteins (“Lipo”) have three N-terminal POTRA domains (**Table S3**), but the presence of a lipid anchor at the N-terminus of the first POTRA domain in 386 out of 513 proteins would attach the domain to the periplasmic surface of either the outer or inner membrane. It is uncertain whether three POTRA domains would be sufficient to span the periplasm in order to allow the lipid to anchor the N-terminus in the inner membrane. Positioning the N-terminal lipid at the periplasmic surface of the outer membrane would fix the POTRA domains: diminishing their flexibility, and serving thereby to constrain exposed regions of the POTRAs to assist interaction with other proteins. These Omp85 lipoproteins are detected in species throughout the Bacteroidetes and Chlorobi, with often more than one copy per genome. Besides BamA and TamA, the Omp85 lipoprotein subfamily is the only group of proteins with a taxonomic distribution indicating vertical inheritance rather than HGT (**Figure 3**).

The Omp85 proteins without any N-terminal extension (“noNterm”; **Figure 1**) might also function in membrane protein biogenesis, given the experimental observation that the mitochondrial homolog of BamA, Sam50, is functional in the binding and the assembly of β-barrel protein substrates into outer membranes even if the single POTRA domain is removed (Stroud et al., 2011). The barrel domains of these proteins show some sequence-based similarities to the Omp85 metalloprotease protein, and could be the ancestor of this subfamily, which subsequently gained the metalloprotease domain (Figures 2A,C; **Table S2**).

The BamA-like proteins are another intriguing subfamily that have 1-3 N-terminal POTRA domains (**Table S3**). They form distinct sequence cluster from the BamA sequences (Figures 2A,C,E; **Table S1**) and are always present in addition to BamA (i.e., each organism with a BamA-like protein also encodes a protein grouped as “BamA” in this study). Based on their barrel+POTRA structure, we hypothesize that these function in a manner similar to BamA and TamA, as membrane protein assembly factors.

The sequence diversity between the subfamilies does not correlate with the taxa in which the sequences are found (**Figure 2B**), supporting that the ten protein subfamilies have ancestries that indicate HGT as well as vertical descent. Investigating the sequence-based similarities on a large scale through visualization of the protein similarity network supported our manual annotation: this is true when considering full-length sequences (**Figure 2B**), when considering only the barrel domain sequences (**Figure 2D**) or N-terminal parts of the sequences (**Figure 2F**), each of which show a consistent clustering of the 10 subfamilies.

### THE TWO-PARTNER SECRETION SYSTEMS: FhaC-TYPE AND Hmw1B-TYPE

The network representation also supports previous observations of a split between two sequence groups of the TpsB proteins, the FhaC subgroup and the Hmw1B subgroup (Jacob-Dubuisson et al., 2013). We observe further differences in the taxonomic diversity of these two TpsB subfamilies: while the FhaC group is comprised almost exclusively of sequences from *Proteobacteria*, the Hmw1B subgroup consists of sequences from a large number of *Cyanobacteria* but also various *Proteobacteria* – in several cases the same taxa encode proteins of the FhaC subgroup as well as the Hmw1B (**Figure 3**).

Domain profiling shows the barrel domain of the Hmw1B subfamily as an Omp85-type barrel in the majority of cases, as opposed to the FhaC group that has the ShlB (TpsB)-type barrel (**Table S2**). However, a structure-based search using Phyre2 confirms that the majority of the Hmw1B proteins are more similar to the FhaC structure, than to the BamA structure (data not shown). The higher sequence similarity to the Omp85-type barrel rather than the TpsB type suggests the Hmw1B group could reflect a more ancestral state and possibly the origin of the TpsB family. This is also in accordance with its taxonomic distribution; the Hmw1B subgroup can be found predominantly in early-branching *Cyanobacteria*, whereas the FhaC-type proteins likely reflect a further level of specification, possibly derived from a gene duplication of an Hmw1B protein and subsequent spread by HGT.

### THE POTRA DOMAINS REVEAL STRIKING SPECIALIZATION

Previous analyses of POTRA sequences showed the sequence relationships between the mitochondrial Sam50 and the plastid Toc75 and Oep80 to proteobacterial and cyanobacterial sequences, respectively (Arnold et al., 2010). We therefore sought to expand this validated approach to use the POTRA domain sequence signatures for an understanding of evolution within the greater Omp85/TpsB superfamily. POTRA domain sequences from TamA, the BamA-like proteins, the Patatin-like sequences, the lipid-anchored BamA-like proteins (Lipo), as well as FtsQ, the only other protein known to encode POTRA domains (Sanchez-Pulido et al., 2003) were collected and compared.

**FIGURE 4:**
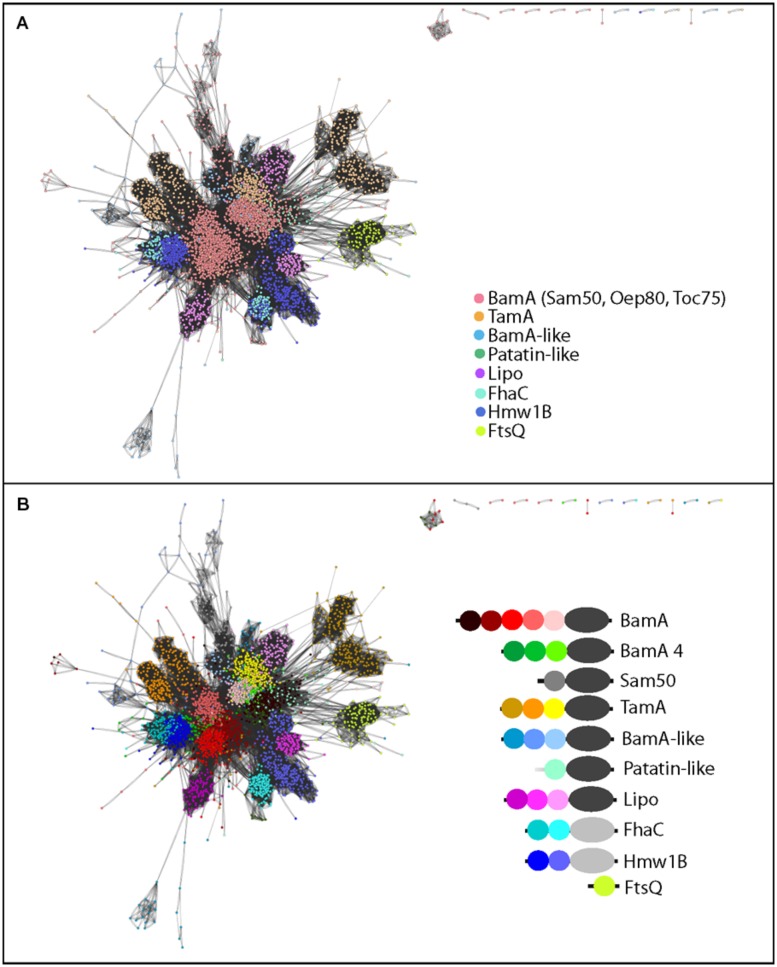
**Sequence similarity network of POTRA domains highlights diversity based on subfamilies as well as the location of the POTRA respective to the barrel. (A)** Protein-protein similarity network representation of POTRA domain sequences, where the colors depict the different subfamilies. **(B)** Recolouring of **(A)** as indicated; with each POTRA domain of each subfamily highlighted in a distinct color. Only the POTRA domains conserved in the majority of the respective sequences (e.g., five for BamA) are shown; for proteins with additional POTRA domains (Arnold et al., 2010), the regions of the five most conserved based on a multiple sequence alignment are depicted, as described in the Section “Methods.”

The POTRA domains of TpsB proteins are so distinct that they conform to a distinct Pfam profile (PF08479 – “POTRA_2”). The majority of POTRA sequences from the Omp85 protein subfamilies conform to Pfam profile PF07244 (“Surf_Ag_VNR”), but even so clear clusters of POTRA sequences are evident (**Figure 4A**). In the case of the TamA protein subfamily and the Omp85-lipoprotein subfamily, the third POTRA domain shows remarkable similarity to the POTRA domains found in BamA, but the first two POTRA domains form discrete clusters. This indicates that while POTRA three is likely directly inherited from the original BamA duplication event leading to the subfamilies, POTRAs one and two have strongly diverged, either through sequence drift or mixing of the secondary structure elements. This fits well with the hypothesis that the POTRA domain closest to the barrel experiences the strongest selective pressure, arising from structural restrictions due to its proximity to the membrane-embedded barrel. Structurally, this POTRA domain makes important contacts with the barrel domain (Noinaj et al., 2013). The distinct features of the more N-terminal POTRAs would be explained by them being the domains that interact with partner proteins, which differ between BamA and TamA (Hagan et al., 2011; Selkrig et al., 2012).

In modular protein complexes, the capacity of binding sites to interact with substrates is often modified by adding or duplicating domains (Bjorklund et al., 2006). The internal POTRA domains (P2-P4) in BamA show highest sequence similarity to each other, consistent with a pattern of domain duplications (**Figure 4B**); and the trend in BamA to duplicate the internal POTRAs goes in accordance with observations on larger scales (Bjorklund et al., 2006). The dynamic potential of POTRA domains is further emphasized by some organisms having BamA sequences with more than five POTRA domains as observed previously (Arnold et al., 2010); only the conserved five POTRAs present in the majority of sequences were included in the analysis (**Figure 4**) to avoid generating too much complexity in the network. The seemingly contrary trend in the TamA and Omp85-lipoprotein subfamilies can be explained by assuming that BamA is the original Omp85, which already carried several POTRA domains, and later functional adaptations led to a divergence of the POTRA domains P1 and P2 in these two subfamilies.

As previously observed, there is complexity within the cyanobacterial BamA cluster, including the plastid Oep80 and Toc75 sequences (Arnold et al., 2010; Koenig et al., 2010). Predominantly, these contain only three POTRA domains, differentiating these sequences from the majority of all other BamA proteins, and some of these POTRA domains conform to the sequence characteristics of TpsB-type POTRAs (**Table S2**, Koenig et al., 2010). For the purpose of the analysis depicted in **Figure 4**, therefore, the entire cluster is colored separately and denoted “BamA 4” (for the fourth largest BamA cluster as given in **Table S1**; **Figure 4B**), consistent with the nomenclature used in **Table S1**. The second POTRA domain (P2) is often recognized by the TpsB-specific POTRA domain motif (PF08479), consistent with previous observations (Arnold et al., 2010). Also of note, BamA from the *Deinococcus-Thermus* phylum, which also clustered in the predominantly cyanobacterial group (BamA4 in **Table S1**), have POTRA P1 domains with strong similarity to the sequence features of the POTRA P2 domain from the FhaC protein subfamily (**Figure 4**). These distinguishing features indicate an adapted function of the BamA of this Phylum, perhaps to unique features of their cell envelope (Farci et al., 2014).

The single POTRA domain for Sam50 is highlighted in gray (**Figure 4B**) and is highly divergent from all bacterial POTRA sequences. This divergence might be a reflection of the simpler substrate repertoire and/or the reduced function of the POTRA domain in the mitochondrial outer membrane, and it is consistent with the observation that Sam50 is functional even if the POTRA domain is deleted (Stroud et al., 2011).

### THE TAXONOMIC DISTRIBUTION OF THE SUBFAMILIES HIGHLIGHTS VERTICAL VERSUS HORIZONTAL INHERITANCE

BamA is essential for outer membrane biogenesis through its catalysis of β-barrel protein assembly. Given the clearly defined “BamA family,” the question of whether a BamA is found ubiquitously in organisms with an outer membrane could be addressed with confidence (**Figure 3A**; **Table S4**). There is no evidence of BamA in genomes from the taxa known to lack a Gram-negative type cell envelope, nor in the proteobacterial obligate intracellular endosymbionts which lack the capacity for outer membrane biogenesis: *Candidatus* Tremblaya princeps; *Candidatus* Hodgkinia cicadicola; *Candidatus* Carsonella ruddii, and *Candidatus* Zinderia insecticola (McCutcheon and Moran, 2012) all lack a gene encoding BamA (**Table S4**, green font). Consistent with this, in the fifth member of the “tiny genome” organisms *Candidatus* Sulcia Muelleri, in which there remains several genes for cell envelope biosynthesis (McCutcheon and Moran, 2012), each of the strains present in our dataset has a BamA sequence (**Table S1**).

We could not identify any BamA proteins for the curious bacterium *Caldisericum exile* (DSM 21853). Electron microscopy shows that *C. exile* has an outer membrane-like envelope, but further experiments failed to clarify whether it is Gram-positive or Gram-negative (Mori et al., 2009); our observation of the lack of BamA or any other proteins annotated as outer membrane-localized (PsortB; Yu et al., 2010) point to *C. exile* having a Gram-positive-type cell envelope.

The distribution of the additional subfamilies is more disseminated. As noted, the Omp85 Lipo in *Bacteroidetes* and *Chlorobi* and TamA in *Proteobacteria* are found in phylogenetic subgroups on phylum-level suggesting their origin from a single BamA duplication followed by vertical inheritance (**Figure 3**; **Table S3**). However, the other Omp85 families indicate a later evolutionary origin in the respective taxa, as they can only be found conserved at genus-level (**Figure 3**; **Table S3**; e.g., Metallo). The latter subfamilies, and this includes FhaC and Hmw1B, show a distribution across a variety of different groups strongly suggesting inheritance through HGT. This mode of inheritance is common for other membrane proteins associated with virulence (Pallen and Wren, 2007), including oligomeric molecular machines such as the protein secretion systems (for example, see Cianciotto, 2005; Alvarez-Martinez and Christie, 2009; Abby and Rocha, 2012). Considerable expansion in diversity has taken place in the *Bacteroidetes*/*Chlorobi* as well as some of the Phyla so far only poorly represented in the sequence databases (*Ignavibacteria, Chrysiogenetes, Verrucomicrobia*), whereas the Phyla considered to be among the early branching ones often encode a single copy of BamA and no other Omp85/TpsB family members (**Figure 3**; *Thermotogae*, *Deinococcus*-*Thermus*).

### A HIGH LEVEL OF DIVERSITY IN BamA, THE Omp85 BLUEPRINT

Given the proposed evolution of Omp85 protein subfamilies from gene duplication events involving BamA, we investigated what appeared to be recent gene duplication events; many organisms were found to have two or more genes encoding BamA paralogs (**Figure 3A**), and phylogenetic analysis of the BamA sequences was used to investigate their evolutionary history. Attempts at aligning the barrel region for all BamA sequences resulted in very few informative sites which could be used for tree calculations. We therefore chose to focus our attention on BamA diversity at a smaller scale, restricted to sequences with higher conservation.

**FIGURE 5:**
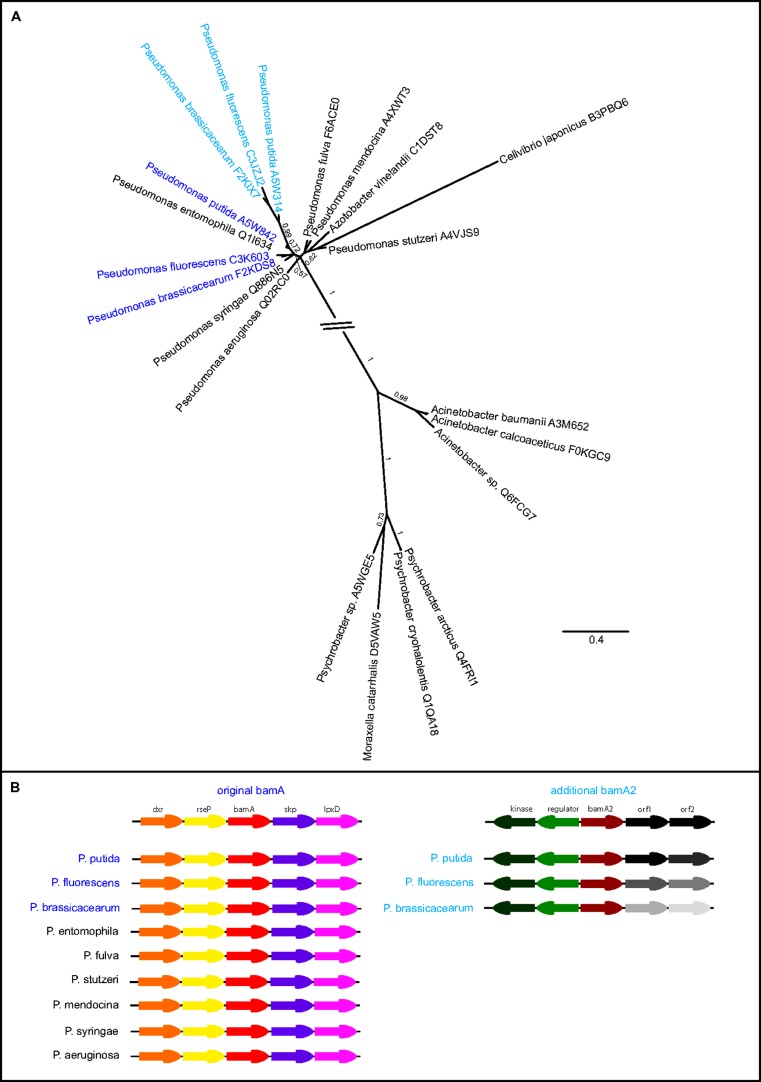
**Highly similar BamA paralogs in *Pseudomonas* spp. (A)** Phylogenetic tree of the *Pseudomonas* spp. BamA sequences and their closest taxonomic relatives. Different colors indicate organisms with more than one BamA copy with dark blue displaying the original (most conserved) sequence, whereas additional copies are displayed in light blue. Tree calculations were performed using phylobayes under the C20 model, posterior probabilities are shown as branch support values. The interrupted branch was shortened for display purposes. **(B)** Synteny view of the *Pseudomonas* spp. bamA and their surrounding genes, the underlying data were retrieved from the NCBI database. The genes upstream of the additional bamA are not conserved, and indicated as “orf1” and “orf2” in the overview and depicted in gray shades in the comparative view.

Several *Pseudomonas* spp. encode two BamA paralogs, and initial sequence alignments showed very high similarity between these BamA sequences and their closest relatives. Phylogenetic analysis of full-length sequences suggested a very recent duplication event resulting in a highly similar duplicate; BamA paralogs are present in non-pathogenic species *P. brassicacearum*, *P. fluorescens* and *P. putida*, which are known for their role in promoting plant growth and bioremediation (**Figure 5A**), and a few other of the numerous sequenced *P. syringae* strains also contain two BamA sequences (**Table S1**). Some species, however, have a single gene encoding BamA; such is the case for strains of the human pathogens *P. aeruginosa* and *P. mendocina* (**Figure 5**; **Table S1**). Analysis of the gene synteny (**Figure 5B**) shows a conserved surrounding of the original *bamA* sequences, whereas the duplicated genes (“*bamA2*”) are at a different location in the genome and share similar downstream genes, whereas the upstream genes differ. This observation confirms our assignment of original versus additional BamA, and also reflects the extremely high genome plasticity in *Pseudomonas* spp. (Silby et al., 2011).

**FIGURE 6:**
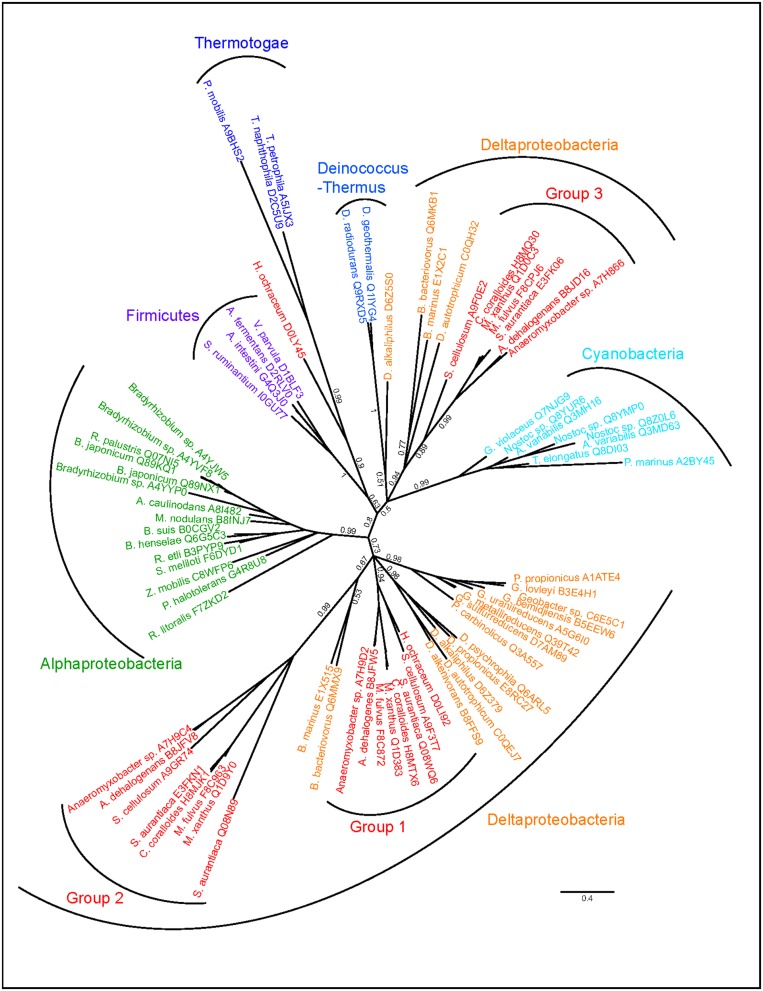
**Independent sets of BamA proteins in the *Myxococcales*.** Phylogenetic tree of BamA sequences identified in *Myxobacteria* and their closest taxonomic relatives. Tree calculations were performed using phylobayes under the C60 model, posterior probabilities are shown as branch support values. The *Myxococcales* Group 1, Group 2, and Group 3 sequences are indicated.

A more complicated scenario is evident in the *Myxobacteria*, which are members of the *Deltaproteobacteria* and are best known for their unusual characteristics such as gliding motility and social behavior (Kaiser, 2003; Nan and Zusman, 2011). BamA paralogs from these species are diverse in copy number (**Figure 3**). Initial sequence alignments indicated that while all belong to the BamA subfamily, three distinct subgroups could be seen with varying numbers of POTRA domains, with some showing similarity to sequences outside the *Deltaproteobacteria*. We therefore used only the sequence corresponding to the barrel domain (see Methods) for the tree inference. To probe for potential HGT events, sequences displaying high similarity to the additional BamA copies were included in the tree calculation alongside BamA sequences from the closest taxonomic relatives. Three distinct monophyletic groupings were evident, each group resulting from one acquisition or duplication event in the *Myxobacteria* and a few close relatives (**Figure 6**). While Group 1 branches according to vertical inheritance, and Group 2 indicates a single duplication within the *Deltaproteobacteria* followed by strong sequence divergence but no HGT, Group 3 seems to have been acquired from one of the early branching phyla (*Firmicutes*, *Thermotogae*, *Deinococcus-Thermus*, *Cyanobacteria*) through HGT. However, given the low sequence coverage of this area of the bacterial tree, as well as the low support for a monophyletic origin with the *Deinococcus-Thermus* and *Cyanobacteria* (branch support 0.5), the exact origin within these phyla should be interpreted with caution. Tree calculations using the C20 model in phylobayes (data not shown) consistently resulted in similar topologies for the monophyly of the *Myxobacteria* Group 1 with the *Deltaproteobacteria* as well as the *Alphaproteobacteria* monophyly, and supports a non-proteobacterial origin of the *Myxobacteria* sequence Group 3, indicating an acquisition through HGT. Group 2 branches off as a monophyletic branch between the *Proteobacteria* and all others possibly reflecting long-branch attraction due to the high divergence of the sequences.

These examples demonstrate the variability of BamA not only in copy numbers, but also in sequence origin and level of similarity. It provides plausibility to the scenario for duplication of BamA genes, followed by selection events for diversification of function. We suggest two scenarios why this selection could be advantageous: (i) the highly similar BamA paralogs (e.g., **Figure 5**) could provide alternatives for control of gene expression, allowing for regulation in response to specific environmental conditions, and (ii) specialization of activity for a certain subset of outer membrane protein substrates, leading ultimately to become modules like TamA that assist the function of the cognate BamA in the assembly of diverse membrane protein substrates (Selkrig et al., 2014).

### POTENTIAL IMPLICATIONS OF DIFFERENCES IN Omp85 PROTEINS

The diversity observed in the Omp85 family could reflect adaptations to different substrate (“client”) proteins, as has been observed in molecular chaperone protein families. Detailed studies on molecular chaperones found in the cytoplasm show high levels of variation with respect to their copy numbers; in order to cope with the assembly of their evolving range of substrate proteins, as well as to acquire novel (sub)functions themselves (Henderson et al., 2013; Ruiz-Gonzalez and Fares, 2013).

Gene duplications for cytoplasmic chaperones such as GroEL (Hsp60), Hsp70 or Hsp90 are very common amongst eukaryotes where the formation of distinct subgroups is well-described (Bogumil et al., 2014), and multiple paralogs of these cytoplasmic chaperones are also observed in prokaryotes (Nimura et al., 2001; Chen et al., 2006; Lund, 2009). For the GroEL-like chaperones, it has been proposed that the initial transfer of specific chaperones between unrelated organisms living in the same environment paves the way for subsequent transfer of other functions important in the respective niche (Williams et al., 2010). The presence of multiple BamA or BamA-like proteins detected through our study might likewise enable the respective organisms to acquire or evolve a more diverse outer membrane proteome, such as the diversity of cytoplasmic chaperones is controlling the mutation rate of proteins, enabling the organisms to generate a more diverse cytoplasmic proteome (Williams and Fares, 2010). This fits with the observations in this study showing that the expansion of paralogs is often specific for certain subgroups or species with a distinct lifestyle, and the enrichment of Omp85 proteins in organisms thriving in less stable environments such as marine or soil bacteria as opposed to pathogens. As the first point of contact, outer membrane proteins play a crucial role in an organism’s interactions with its surroundings; the gain of specific Omp85 subfamilies could mediate adaptation on a rapid scale.

## SUMMARY

The protein architecture and sequence signatures identified within the Omp85/TpsB superfamily enables a classification structure to this highly diverse group of proteins. It suggests that the complex process of assembling proteins into bacterial outer membranes selects for diversity in the genes encoding BamA paralogs and BamA-related functions. Beyond the established and ancient BamA protein subfamily, other Omp85 protein subfamilies are present and have been acquired through HGT to become established in diverse bacterial taxa. We suggest that proteins with a barrel+POTRA domain architecture or the barrel-only Omp85 proteins serve as accessory modules in the β-barrel assembly machinery: assisting BamA to assemble subsets of outer membrane proteins, thereby enabling acquisition of a range of new genes for outer membrane proteins to be acquired. This diversity in Omp85 proteins thereby provides the potential for the organism to thrive in a new or changing environment.

## AUTHOR CONTRIBUTIONS

Eva Heinz and Trevor Lithgow conceived the study. Eva Heinz designed and performed the experiments and analyzed and interpreted the data. Eva Heinz and Trevor Lithgow wrote the manuscript.

## Conflict of Interest Statement

The authors declare that the research was conducted in the absence of any commercial or financial relationships that could be construed as a potential conflict of interest.

## Footnotes

1 http://www.r-project.org/

2 http://pfam.sanger.ac.uk; version 27

3 http://supfam.org/SUPERFAMILY/cgi-bin/genome_names.cgi

## SUPPLEMENTARY MATERIAL

Table S1 **List of all UniProt accession numbers of Omp85 proteins in their respective clusters**.

Table S2 **List of the domain profiles identified for the main clusters based on the annotation of Pfam domains in Interpro**.

Table S3 **Summary of the prediction results using Phyre2 of sequences with novel domain profiles**.

Table S4 **List of all bacterial species with a completed proteome according to the UniProt database at the time of analysis, which lack a protein similar to BamA.** Organisms that represent exceptions (highly reduced obligate intracellular bacteria, organisms with indications for Gram-positive or Gram-negative cell envelope) are highlighted in green, organisms where a BamA would be expected due to its presence in all other strains of the respective species are highlighted in red. All taxa underlined in gray are described to display a Gram-positive cell envelope.
